# Posterior Cruciate Ligament reconstruction with peroneus longus tendon graft: 2-Years follow-up

**DOI:** 10.1016/j.amsu.2019.05.009

**Published:** 2019-05-31

**Authors:** Riky Setyawan, Noha Roshadiansyah Soekarno, Asa Ibrahim Zainal Asikin, Sholahuddin Rhatomy

**Affiliations:** a Soeradji Tirtonegoro Sport Center and Research Unit, Dr Soeradji Tirtonegoro General Hospital, Klaten, Indonesia; b Department of Orthopaedics and Traumatology, Dr Soeradji Tirtonegoro General Hospital, Klaten, Indonesia

**Keywords:** Posterior cruciate ligament, PCL reconstruction, Peroneus longus tendon, Autograft

## Abstract

**Background:**

Several study that evaluate the usage of peroneus longus tendon (PLT) autograft in Anterior Cruciate Ligament reconstruction shows good result. Regardless the potential, there was no study about the use of PLT autograft in Posterior Cruciate Ligament (PCL) reconstruction. The purpose of this study is to evaluate the functional outcome and donor site morbidity after single bundle PCL reconstruction using PLT autograft.

**Methods:**

Patient who met inclusion criteria, enrolled to this study and underwent single bundle PCL reconstruction using PL tendon autograft. Clinical outcomes were assessed with International Knee Documentation Committee (IKDC), Modified Cincinnati scoring systems, Lysholm score, and Serial hop test (single hop test and triple hop test) 2-year after surgery. Donor site morbidity was assessed with Foot and Ankle Disability Index (FADI) and American Orthopaedic Foot and Ankle (AOFAS) scoring system.

**Results:**

Fifteen patients fulfilled the inclusion criteria (11 males and 4 females). PLT graft diameters were 7.5–10 mm (mean: 8.30 ± 0.65 mm). Significant increase of functional score (*p* < 0.05) were found two years after surgery. Mean score of IKDC was 47.58 ± 11.75 pre-operative; 78.17 ± 4.52 post-operative, Modified Cincinnati was 48.86 ± 12.22 pre-operative; 79.00 ± 4.82 post-operative, Lysholm score was 49.26 ± 11.54 pre-operative; 80.20 ± 5.04 post-operative. FADI and AOFAS at donor site ankle was 93.00 ± 3.04 and 93.26 ± 4.20, respectively. Serial hop test showed good result.

**Conclusion:**

PCL reconstruction using peroneus longus tendon autograft shows good functional outcome of the knee based on IKDC, Modified Cincinnati, Lysholm score, with preservation of ankle function based on AOFAS and FADI score at 2-years follow-up.

## 1 Introduction

The incidence of isolated Posterior Cruciate Ligament (PCL) in acute knee injuries is low. Ruptured PCL can result in abnormal knee kinematics and increase the risk of subsequent injury to other knee ligament. Nonsurgical treatment is considered appropriate in patient with isolated grade I, II, or grade III patients with mild symptoms and low demand. Surgical treatment with PCL reconstruction is the treatment of choice in patient with symptomatic grade III PCL injury or if concomitant injuries to other knee ligament occur (1).

Different type of autografts have been studied in PCL reconstruction, with hamstring autograft being one of the most common graft used. Hamstring autograft is easy to harvest, and has less donor morbidity compared to Bone-Patellar-Tendon-Bone (BPTB) autograft. While the use of BPTB may allow faster return to sport, it also carries potential disadvantages of anterior knee pain, kneeling pain, and loss of motion (2). The disadvantages of hamstring tendon are unpredictable graft size, potential reduction of hamstring muscle power, and thigh hypotrophy (3). Presence of these disadvantages limit the use of hamstring autograft in athlete who need dominant hamstring power to compete at the highest level. Moreover, even though the incidence of anterior knee pain and kneeling pain after hamstring graft harvesting is fewer than BPTB, if present, it can be very disturbing, especially in Asians who frequently kneel as part of their daily activity. For this particular group of patients, some author tried to evaluate the use of other type of autograft, including the use of Peroneus longus tendon (PLT) autograft.

Peroneus longus tendon autograft was already used in some orthopaedic procedures [4,5]. Some authors also have studied its use in ACL reconstruction (6–8), with most of the studies show good clinical result and minimal donor site morbidity of the harvested ankle. Previous biomechanical study also showed that peroneus longus tendon autograft have comparable tensile strength compared to hamstring tendon [9]. The result of the study mentioned above showed that PLT is a potential graft of choice in knee ligament reconstruction because of the overall good clinical result, low ankle donor site morbidity, no knee donor site morbidity, and comparable tensile strength compared to more popular hamstring tendon. Regardless of the potential, no previous study evaluate the use of PLT autograft in PCL reconstruction.

The purpose of this study was to evaluate the functional outcome and donor site morbidity of PCL reconstruction with peroneus longus tendon autograft in 2-year follow up.

## 2 Materials and methods

This study was a prospective cohort study with consecutive sampling of patient with PCL injury who underwent PCL reconstruction between October 2015 and June 2016. The diagnosis of chronic ligament rupture was established with clinical examination and imaging (Magnetic resonance imaging, MRI). The following inclusion criteria were adopted [1]: chronic injury (>6 months) [2], presence of an ‘isolated’ PCL lesion [also including the presence of slight varus/valgus instability (1+) compared with the contralateral limb] and [3] no previous ligamentous surgery. The presence of a posterolateral and/or posteromedial lesion was excluded by clinical examination alone. Fifteen patients fulfilled these criteria and were included in this study. Mean age at the time of injury was 25.86 ± 6.46 years (range 18–38 years). The group consisted of 11 men and 4 women. All patients had chronic injuries with a mean time from original injury to reconstruction of 8 months (range 6–24 months). The injuries occurred during a sports activity in 8 patients (6 soccer, 2 other sports) and during a motor vehicle accident in 7 patients. See Table 1. At arthroscopy examination, a medial meniscal tear was detected in 2 patients, and a lateral meniscal tear in 1 patient and were excluded from this study. This research work has been reported in line with the STROCSS statement [10].

**Table 1:** Subjects characteristics.

Characteristics	Mean	SD	Min	Max	N
AGE	25.86	6.46	18.00	38.00	
SEX					
MALE					11 (73.3)
FEMALE					4 (26.7)
SITE OF INJURY					
DEXTRA					8 (53.3)
SINISTRA					7 (46.7)
INJURY MECHANISM					
TRAFFIC INJURY					7 (46.7)
SPORT					8 (53.3)
GRAFT DIAMETER	8.30	0.65	7.50	10.00	

The functional score of the patient were assessed before the surgery and 2-year after the surgery with International Knee Documentation Committee (IKDC) score, Modified Cincinnati Rating System, Lysholm scale, and serial hop test. Donor site morbidity were assessed with American Orthopaedic Foot Ankle Society score and Foot Ankle Disability Index. Thigh circumference measured in 10 cm and 20 cm superior to upper pole of patellar bone in injury site and contralateral healthy site.

### 2.1 Statistical analysis

Lemeshow method was used to calculate sample size. Method is shown below:

The proportion of patient with PCL rupture (p) was found to be around 6% in our study population. With 95% CI, and precision level of 10%, the calculation were (1.96)^2^ x 0.06 x (0.94)/(0.1)^2^ = 24 samples. There were at least 24 patients in each group needed to be included in this study.

The outcomes of continuous measurements (IKDC, Modified Cincinnati, and Lysholm score) were compared between the 2 groups using the Mann-Whitney *U* test. Statistical significance was accepted at *p* < 0.05. According to this sample calculation, we should enrolled minal 24 patients in one year. In this study, we only took 5 months (October 2015 until February 2016) for patient enrollment.

### 2.2 Surgical technique

A single senior knee surgeon performed all of the PCL reconstruction procedures. The procedure was performed under general anesthesia with the patient in supine position. After brief clinical examination under anesthesia, padded tourniquet was applied in proximal thigh of the affected knee. Anterolateral and anteromedial arthroscopic portals were used for diagnostic arthroscopy. After the diagnosis of PCL rupture is confirmed arthroscopically, PLT autograft was harvested from the ipsilateral ankle.

**Fig. 1:**
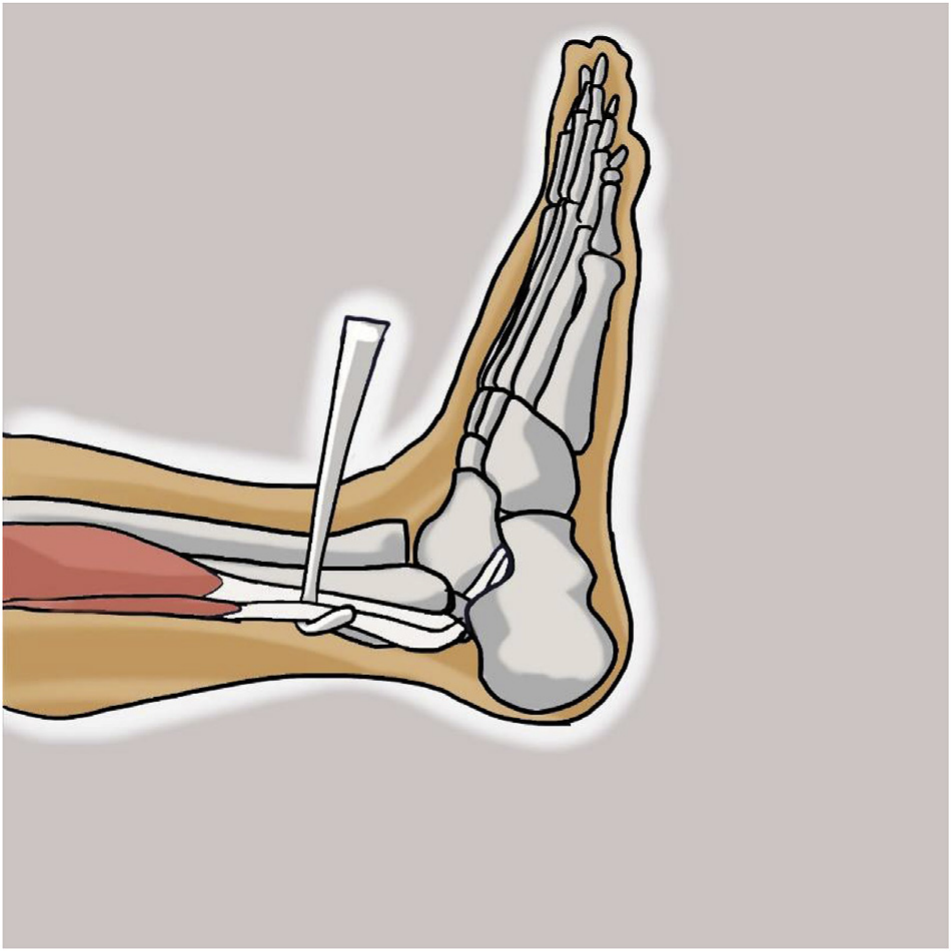
Identification of peroneus longus and peroneus brevis tendon.

**Fig. 2:**
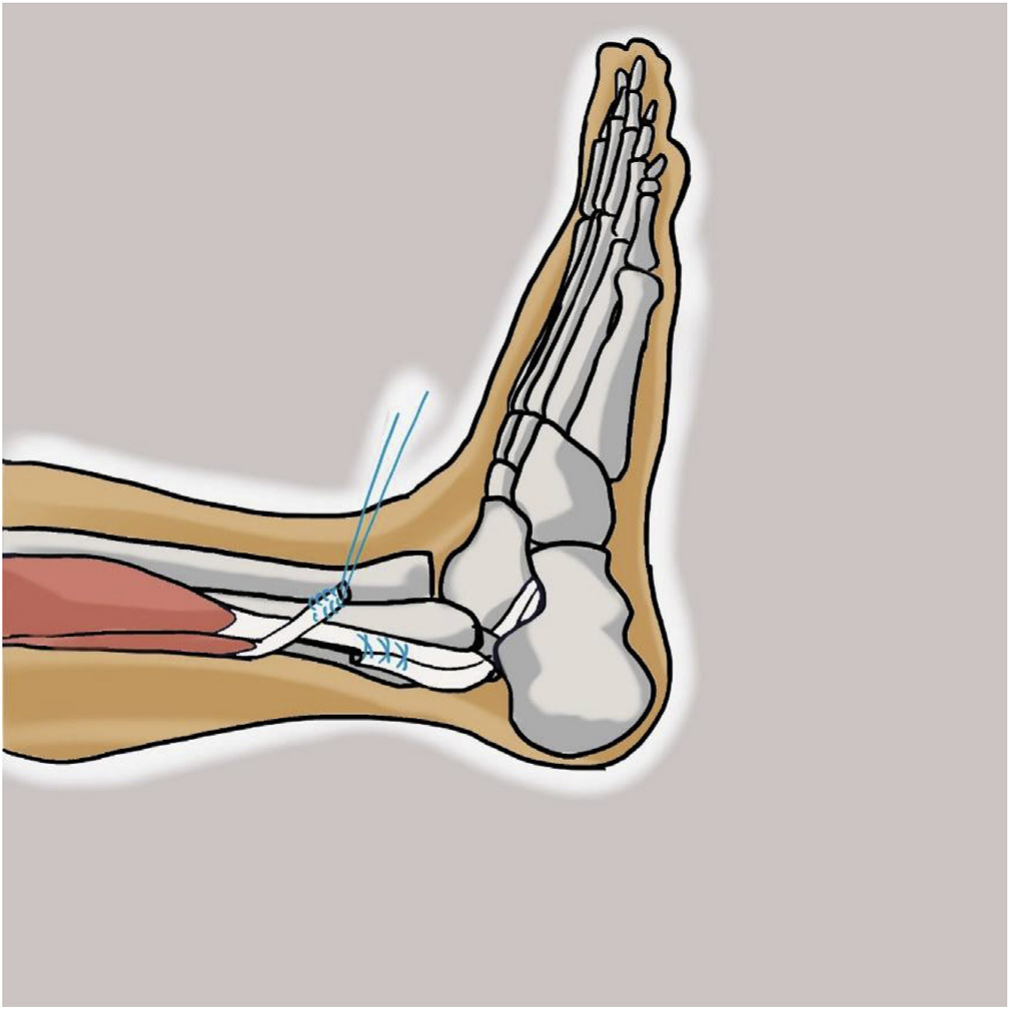
Tenotomy of peroneus longus tendon.

**Fig. 3:**
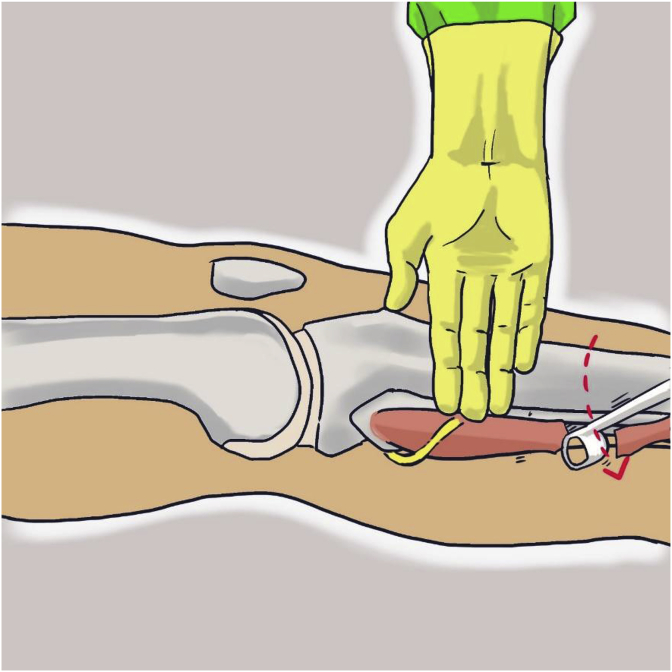
Marked the end of peroneus longus tendon harvest at three fingers bridge below the tip of fibular head.

With the knee in full extension, an approximately 3-cm longitudinal incision was made approximately 2–3 cm above and 1 cm behind the lateral malleolus. The incision was carried through the skin and subcutaneous tissue. After incision of the superficial fascia, peroneus longus and peroneus brevis tendon were identified in the surgical field. After division of the peroneus longus tendon 2–3 cm proximal to the lateral malleolus, the distal part of the tendon was sutured to the peroneus brevis tendon with side to side suture (Fig. 1, Fig. 2). Then the peroneus longus tendon was stripped proximally with a tendon stripper and stopped at the level of 4–5 cm from the fibular head to prevent peroneal nerve injury (Fig. 3).

Synovial and fat-like tissue on the femoral attachment of the PCL remnant was removed carefully to expose the fibers of PCL bundles. The PCL remnants were preserved. The femoral tunnel was placed at 8–10 mm from anterior or distal medial femoral articular margin on a continuous line with the junction of the roof and medial wall of the intercondylar notch. A 2.0 mm Kirschner wire was inserted through the reamer as a guide wire. Over drilling was done with a 5 mm diameter drill (ConMed^©^, USA) using the anterolateral portal. A 2.4-mm pin passed through the femoral tunnel, and reamed using cannulated drill in accordance with graft diameter at the distal portion until 30 mm depth of femoral tunnel.

A posteromedial portal was created under direct vision. The PCL tibial attachment site was completely exposed. A guide pin was inserted through the anteromedial incision within the distal center portion of tibial insertion of PCL, which comes into contact with the posterior edge of retrospinal surface. The tibial hole was made in accordance to graft diameter. A 2.4-mm (blunt leading end) pin was inserted through this hole. A pullout suture was threaded in a retrograde fashion. Using this, the 4-strand hamstring graft pulled through the femoral hole. Proximal femoral fixation obtained with a button (Graftmax^®^, ConMed^©^, USA). Button was flipped outside the medial cortex of the femur. Then, graft was grasped and pulled tightly out of the anterior tibial hole, and a 25–35 mm bioabsorbable screw (BioScrew**®,** ConMed^©^, USA) was inserted at 90° knee flexion while maintaining anterior drawer pull of the tibia.

### 2.3 Postoperative rehabilitation

The knee was immobilized for 4 week with brace in full extension. Ambulation with non-weight bearing protocol was initiated on the second postoperative day. Quadriceps isometric exercise, and straight-leg raising exercise initiated after 2 week. Protected ROM was gradually increased from 0 to 90° flexion starting from the fourth week. After 8 weeks, progressive knee flexion from 90° to full ROM was exercised gradually. Partial weight bearing was permitted after 4 weeks. Full weight bearing with hamstring-strengthening exercises was permitted after 8 weeks and active knee ROM should progress to complete flexion and extension. Patients usually returned to their normal daily activity and were allowed to exercise on a stationary bike or standing on a single leg starting at 5 months postoperatively. Light sports activities began at 6 months. After 12 months, the patients is evaluated with serial hop test (single hop test and triple hop test) and then cleared for sport activities if the result is good.

### 2.4 Clinical and functional evaluation

Post-operative functional outcome (IKDC, Modified Cincinnati, and Lysholm) were recorded two years after surgery with direct patient examination and a guided-interview by a single orthopaedic surgeon outside the surgical team. Donor site morbidity was evaluated with measurement of ankle functional score using AOFAS and FADI score. Serial hop test was assessed at two-years after surgery.

This study was reviewed and approved by the Medical and Health Research Ethics Committee at the Faculty of Medicine of Universitas Gadjah Mada (IRB number KE/1275/11/2018). This study has been registered in a publicly accessible database and having a unique identifying number: researchregistry4760.

### 2.5 Statistical analysis

Statistical data was analysed by an independent statistician. Paired *t*-test was used for comparisons of IKDC, Modified Cincinnati, and Lysholm score from preoperative assessment to 2 years follow-up. Statistical significance was accepted at a *p-*value of <0.05. FADI, AOFAS, and serial hop test (single hop test and triple hop test) were shown descriptively.

## 3 Results

During the period of the study, eighteen patients underwent single bundle PCL reconstruction. Three patients were excluded because of concomitant meniscal injury. Fifteen patients fulfilled the inclusion criteria and underwent single bundle PCL reconstruction with peroneus longus autograft. At 2 years follow up, there were fifteen patients which consist of 11 males and 4 females.

### 3.1 Graft diameter

Intraoperatively, graft diameter was measured and recorded, with the result shows mean diameter of 8.30 ± 0.65 mm (range 7.5–10 mm). See Table 1.

### 3.2 Functional outcome

There were significant differences between the preoperative and 2-year postoperative score in IKDC, Modified Cincinnati, and Lysholm score (*p* < 0.05), as shown in Table 1. Mean IKDC score was 47.58 ± 11.75 (range 32.20–66.70) pre-operatively and 78.17 ± 4.52 (range 72.40–86.20) at 2 years follow-up. Mean score of Modified Cincinnati was 48.86 ± 12.22 (range 34.00–68.00) pre-operatively and 79.00 ± 4.82 (range 72.00–88.00) at 2 years follow-up. Mean Lysholm score was 49.26 ± 11.54 (range 35.00–68.00) pre-operatively and 80.20 ± 5.04 (range 72.0–90.0) at 2 years follow-up. There were significant differences between pre-operative and 2 years post-operative score in IKDC, Modified Cincinnati, and Lysholm tests (*p* < 0.05) with majority of the patient with PCL injury reconstructed with peroneus longus tendon had improvement results. See Table 2. Single hop test and triple hop test after 24 months post operatively shown 95.73 ± 3.08 and 91.86 ± 1.92, respectively. See Table 3.

**Table 2:** Functional outcome.

	PRE-OPERATIVE			POST-OPERATIVE			SIGNIFICANCY
	MEAN	SD	NORMALITY	MEAN	SD	NORMALITY	
IKDC	47.58	11.75	0.072	78.17	4.52	0.164	0.000
MODIFIED CINCINNATI	48.86	12.22	0.024	79.00	4.82	0.549	0.001
TEGNER-LYSHOLM	49.26	11.54	0.056	80.20	5.04	0.587	0.000

**Table 3:** Donor site morbidity and serial hop test.

	MEAN	SD	MIN	MAX	NORMALITY
FADI	94.80	2.42	90.00	100.00	0.900
AOFAS	94.46	2.56	90.00	100.00	0.523
SINGLE HOP	95.73	3.08	90.00	100.00	0.553
TRIPLE HOP	91.86	1.92	88.00	96.00	0.652

### 3.3 Thigh circumference

Result of thigh circumference revealed no difference between injury site and contralateral healthy site (*p* > 0.05). The mean circumference in 10 cm proximal to upper patellar bone was 42.73 ± 4.33 at injury site and 43.83 ± 4.27 at contralateral healthy site. The mean circumference in 20 cm proximal to upper patellar bone was 49.67 ± 4.60 at injury site and 50.40 ± 4.66 at contralateral healthy site. See Table 4.

**Table 4:** Thigh circumference.

	INJURY SITE					CONTRALATERAL SITE					SIGNIFICANCY
	MEAN	SD	MIN	MAX	NORMALITY	MEAN	SD	MIN	MAX	NORMALITY	
10CM THIGH DIAMETER	42.73	4.33	36.50	53.00	0.181	43.83	4.27	37.0	54.00	0.243	0.490
20CM THIGH DIAMETER	49.67	4.60	42.00	60.00	0.933	50.40	4.66	41.50	61.00	0.970	0.668

### 3.4 Donor site morbidity

For the evaluation of donor site morbidity, ankle functional score is measured with AOFAS and FADI score. The mean of AOFAS score of donor ankle was 94.46 ± 2.56 (range 90.0–100.0) and FADI score was 94.80 ± 2.42 (90.0–100.0). See Table 3.

## 4 Discussion

Nonoperative treatment in high grade PCL injury might cause deterioration of knee function with increasing time-lapse after the injury. The deterioration might occurs in less than 5 years after the initial injury based on radiographic finding, patient's subjective symptoms, and functional criteria [11]. Therefore, in this group of patient, reconstruction of the PCL is advocated to return the normal kinematic of the knee and prevent deterioration of knee function.

Previous study have reported that the outcome of PCL reconstruction procedure is influenced by many factors, among the important factors to be considered are graft fixation technique, arthroscopic portals used, bundles addressed, and choice of graft used. Among these factors, choice of graft is one of the most important consideration because it is related to the unique characteristic of each graft. Some graft may be more suitable to certain group of patient related to its advantages, while other type of graft may be more suitable for other related to the potential donor site morbidity. Overall, the consideration for choice of graft in PCL reconstruction is almost the same with ACL, however, the clinical outcome of this two different procedure might be not comparable each other [12].

Two of the most widely used graft in PCL reconstruction are BPTB autograft and hamstring tendon autograft. Compared to other type of graft, BPTB have the advantage of faster return to sport related to its bone-to-bone tunnel healing. The disadvantage of BPTB including presence of tendon proliferation and fat pad fibrosis that can result in infrapatellar contracture syndrome [13]. BPTB harvesting also carries substantial risk of anterior knee pain, kneeling pain, loss of motion [2], and risk of patellar fracture [14].

Presence of kneeling pain or anterior knee pain might be more disturbing in certain group of patient than other. This is especially true in group of patient who frequently kneel as part of their daily activity, whether it is related to religious activity or tradition. Corry et al. [15] compared the clinical outcome and donor site morbidity of patient with isolated ACL rupture who underwent ACL reconstruction with hamstring tendon and patellar tendon. From the patellar tendon group, about 55% patient had anterior kneeling pain in the first year that improved to about 31% in the second year, compared to just 6% in the hamstring group both in the first and second year. A meta-analysis in 2015 by Xie et al. [2] compared the use of BPTB and hamstring tendon autograft, this study showed that the risk ratio for anterior kneeling pain was 1.71 in favour of hamstring tendon, while the risk ratio for kneeling pain was 2.05, also in favour of hamstring tendon. While the percentage of kneeling pain is considered low in the hamstring group compared to the BPTB group, it can be catastrophic if it occur in patient who could not tolerate any kneeling pain.

Hamstring tendon autograft has many advantages compared to BPTB and is gaining popularity in PCL reconstruction. The quadrupled graft or double loop of semitendinosus and gracilis tendons has greater strength than BPTB [16,17]. The use of hamstring tendon in PCL reconstruction also showed good clinical outcome. Chan et al. [18] evaluated the clinical outcome of PCL reconstruction with hamstring tendon during 3–5 years follow up and found significant improvement in knee function, activity level, IKDC classification, Lysholm scores, and muscle strength. Some disadvantages of hamstring tendon autograft harvesting including saphenous nerve injury, thigh hypotrophy, and hamstring muscle power reduction [3,13,19]. Some study also showed inconsistent graft diameter after hamstring tendon harvesting, with most of times the diameter is too small. The relatively small diameter of hamstring tendon might predispose to increased rerupture rate and revision rate after PCL reconstruction. Recent biomechanical study showed that hamstring graft with diameter of 6 mm or 7 mm have significant lower load to failure compared to graft with greater diameter, this study also mentioned a possibility that hamstring graft may not be as strong as previously appreciated in older study [20]. These disadvantages drove some author to evaluate the use of other source of autograft as an alternative to hamstring graft in cruciate ligament reconstruction.

Previous biomechanical study that compared the tensile strength of peroneus longus tendon, hamstring tendon, patellar tendon, and quadriceps tendon showed that the tensile strength of peroneus longus was comparable to hamstring tendon, and was significantly stronger than patellar tendon and quadriceps tendon [21]. Some clinical study also showed good clinical result in the use of peroneus longus tendon in ACL reconstruction [[6], [7], [8]] while other author already used PLT in PCL reconstruction [22].

In this study, we found that the mean of peroneus longus graft diameter was 8.30 ± 0.65 mm (range 7.5–10 mm). Previous study stated that graft diameter of more than 8 mm had lower failure rate in ACL reconstruction, with the likelihood of revision rate was 0.82 lower with increasing 0.5 mm in range 7.0–9.0 graft diameter [23].

The result of this study showed that PCL reconstruction with PLT had significant improvement with good clinical outcome in 2-year follow up based on IKDC, Modified Cincinnati, and Lysholm scores. This result shows that PLT autograft can be used in single bundle PCL reconstruction with good functional outcome of the patient at 2 years follow up. Test for evaluating knee function using single hop test and triple hop test also show good results, which is greater than 90%.

Angthong et al*.* [8] stated that there were some possible donor site morbidity with peroneus longus tendon harvesting. The potential donor site morbidity including ankle function deterioration and concern of ankle instability. In this study, ankle function is measured with AOFAS and FADI score. The result shows that the function of donor ankle was excellent even after harvesting of peroneus longus tendon. This finding is probably related to intact peroneous brevis muscle that will maintain ankle eversion function. Previous study shows peroneus brevis as a more potent ankle evertor that maintain eversion power of the ankle after peroneus longus harvesting [24].

The limitations of this study is limited sample size and absence of objective measurement of ankle eversion strength and objective measurement of knee laxity during two years evaluation. However, some of the potential bias is limited by using a single surgeon, same rehabilitation protocol, and also same surgical procedure in all patients.

With the result of this study, the use of peroneus longus as graft of choice in single bundle PCL reconstruction can be encouraged in clinical practice because it shows good functional score and minimal donor site morbidity.

## 5 Conclusion

Single bundle PCL reconstruction with peroneus longus tendon autograft had improvement functional outcome (IKDC, Modified Cincinnati, Lysholm) and shown excellent ankle function and serial hop test result at two-years evaluation.

## Data availability

The data used to support the findings of this study are available from the corresponding author upon request.

## Provenance and peer review

Not commissioned, externally peer reviewed.

## Ethical Approval

The informed consent form was declared that patient data or samples will be used for educational or research purposes. Our institutional review board also provide an ethical approval with KE/1275/11/2018 as the protocol number.

## Sources of funding

The authors declare that this study had no funding resource.

## Author contribution

Riky Setyawan and Sholahuddin Rhatomy conceived the study. Sholahuddin performed surgery, collected and analysed data. Riky Setyawan drafted the manuscript, analysed data, and critically revised the manuscript for important intellectual content. Noha Roshadiansyah Soekarno and Asa Ibrahim Zainal Asikin analysed data, prepared and drafted the manuscript. Riky Setyawan and Sholahuddin Rhatomy reviewed and edited manuscript. Riky Setyawan and Sholahuddin Rhatomy facilitated all project-related tasks.

## Conflicts of interest

No potential conflict of interest relevant to this article was reported.

## Trial registry number

None.

## Guarantor

Sholahuddin Rhatomy, M.D.

## Registration of research studies

This study has been registered in a publicly accessible database and having a unique identifying number: researchregistry4760.

## Consent

Written informed consent was obtained from the all of the patients for publication of this case report and accompanying images. A copy of the written consent is available for review by the corresponding author of this journal on request.
